# Corticosteroid Burden and Hearing Outcomes in Eosinophilic Otitis Media with and without Biologic Therapy: A Pilot Retrospective Cohort Study

**DOI:** 10.1159/orl/aegag008

**Published:** 2026-10-15

**Authors:** Takashi Wada, Yasuhiro Arai, Yoshihiro Aizawa, Kiyoshi Sakasai, Hiromitsu Hatakeyama, Nobuhiko Oridate

**Affiliations:** a Department of Otolaryngology and Head and Neck Surgery, Yokohama City University Medical Center, Yokohama, Japan; b Department of Otolaryngology and Head and Neck Surgery, Yokohama City University School of Medicine, Yokohama, Japan

**Keywords:** Eosinophilic otitis media, Biologics, Dupilumab, Mepolizumab, Eosinophilic chronic rhinosinusitis

## Abstract

**Introduction:**

Eosinophilic otitis media (EOM) is a refractory, type 2-driven form of otitis media that often requires repeated local and/or systemic corticosteroid therapy. Biologic agents prescribed for comorbid type 2 airway disease may improve EOM outcomes; however, comparative real-world data remain limited. Therefore, we aimed to evaluate changes in EOM severity, air-conduction pure-tone average (PTA), and corticosteroid treatment burden in patients with EOM treated with and without biologic therapy in a single-center retrospective cohort.

**Methods:**

We retrospectively reviewed consecutive patients with EOM treated at a single tertiary center. The index date was defined as initiation of biologic therapy (dupilumab or mepolizumab) in the biologics group and initiation of EOM-directed corticosteroid therapy (systemic and/or intratympanic) in the non-biologics group. Post-index outcomes were assessed within a prespecified 10- to 14-month window, selecting the visit closest to 12 months. The primary analysis was conducted at the patient level, averaging bilateral ear outcomes when applicable. Primary outcomes were the EOM severity score (0–10) and air-conduction PTA (0.5/1/2/4 kHz).

**Results:**

Fourteen patients (24 ears) were included (mean age: 57.3 ± 9.3 years; three men/11 women)in this study, with 7 patients per group. Follow-up timing was standardized (median: 12.5 months; interquartile range [IQR]: 12.0–13.0). Baseline severity score and air-conduction PTA differed between groups (4.9 ± 1.7 vs. 5.7 ± 1.1; 39.6 ± 19.0 vs. 55.0 ± 19.0 dB, respectively). Severity improved in both groups; however, the between-group difference in change did not reach statistical significance (*p* = 0.077). Air-conduction PTA improved within the biologics group (*p* = 0.031); however, the between-group difference in PTA change was not significant (*p* = 0.249). Systemic corticosteroids were discontinued in the biologics group (0.00 ± 0.00 mg/day) but remained required in the non-biologics group (1.35 ± 1.55 mg/day; *p* = 0.011).

**Conclusion:**

In this pilot real-world cohort, biologic therapy used for comorbid type 2 airway disease was associated with a reduced corticosteroid burden and lower post-index EOM severity. Air-conduction PTA improved within the biologics group; however, the between-group difference in PTA change was not statistically significant, and the hearing findings should therefore be considered exploratory. These preliminary findings should be interpreted cautiously and may help inform adequately powered future studies.

## Plain Language Summary

Eosinophilic otitis media (EOM) is a long-lasting form of middle-ear inflammation in which a type of white blood cell called an eosinophil plays an important role. People with EOM often also have asthma or severe sinus disease, and they may need repeated steroid treatment by mouth or injections through the eardrum. Steroids can help symptoms, but repeated or long-term use can cause side effects, so treatments that reduce steroid use are important. We reviewed the medical records of 14 people with EOM treated at one hospital. Seven patients received biologic medicines, which are targeted treatments used for asthma or sinus disease, and 7 patients were treated without biologics. We compared ear disease severity, hearing test results, and steroid use before treatment and about 1 year later. Patients who received biologics stopped using systemic steroids after treatment, while patients treated without biologics still needed some steroid treatment. The biologics group also needed fewer steroid injections into the ear and had lower ear disease severity after treatment. Hearing improved within the biologics group, but the difference in hearing change between the two groups was not clear. Because this was a small study based on past medical records and the two groups were not fully comparable at the start, the findings should be interpreted carefully. The findings suggest that biologics may help reduce steroid burden in selected patients with EOM, but larger planned studies are needed.

## Introduction

Eosinophilic otitis media (EOM) is a refractory form of otitis media characterized by eosinophil-dominant middle-ear effusion and frequent comorbidity with bronchial asthma and eosinophilic chronic rhinosinusitis (ECRS), reflecting a type 2-driven inflammatory phenotype [1–4]. In a systematic review, hearing loss was the most common otologic manifestation (approximately 65%), and a large proportion of patients had asthma (approximately 95%) and nasal polyposis (approximately 71%), underscoring the close clinical linkage between middle-ear and airway disease [3].

The clinical burden of EOM is significant because auditory dysfunction can progress and may be difficult to reverse. Conductive hearing loss may evolve into mixed hearing loss with sensorineural deterioration over time; bone-conduction thresholds have been reported to worsen in approximately half of patients, and complete deafness in a minority (approximately 6%), occasionally prompting consideration of advanced hearing rehabilitation (e.g., cochlear implantation) [2, 4]. Delayed recognition may lead to repeated ineffective treatments, persistent otorrhea or effusion, and cumulative impairment in quality of life [3].

Anti-inflammatory therapy remains the mainstay of care; however, the steroid burden is often significant. In the same systematic review, intratympanic steroid injections and systemic corticosteroids were used in approximately 39% and 33% of reported cases, respectively, reflecting frequent reliance on local and/or systemic corticosteroid strategies in real-world management [3]. Importantly, “successful treatment” in the literature is often defined in part by decreased steroid dependence – sometimes requiring long-term periodic or continuous administration – highlighting the practical challenge of tapering therapy in steroid-responsive but relapsing disease [3]. Given the well-described harms associated with even short courses of oral corticosteroids in general adult populations, steroid-sparing approaches are clinically attractive [5].

Biologic therapies targeting type 2 inflammation have become widely used for severe asthma and ECRS, and selected agents may plausibly improve EOM by suppressing eosinophilic inflammation across the unified airway and middle ear. In this study, we specifically evaluated dupilumab (anti-interleukin-4 receptor alpha [anti-IL-4Rα]) and mepolizumab (anti-IL-5), which case reports and small observational studies have reported to improve EOM-related clinical outcomes [6–9]. Other biologics, such as anti-IgE omalizumab and anti-IL-5Rα benralizumab, have also been reported in EOM, although the evidence remains limited [10–12]. More recent observational cohorts have evaluated biologics in refractory EOM associated with asthma and chronic rhinosinusitis with nasal polyps over relatively short follow-up periods [13, 14]. However, the current evidence base remains limited and heterogeneous, and meta-analytic data suggest that while biologics may improve symptom- or severity-related measures, consistent improvement in audiometric hearing thresholds has not been clearly demonstrated, emphasizing the need for more rigorous comparative real-world analyses [3, 15].

Therefore, we aimed to evaluate changes in EOM severity, air-conduction hearing outcomes (including pure-tone average [PTA]), and corticosteroid treatment burden in patients with EOM treated with and without biologic therapy in a single-center retrospective cohort, focusing on quantitative metrics (average daily systemic corticosteroid dose and intratympanic injection frequency). To reduce clustering-related bias, we prespecified patient-level analyses (ear-averaged outcomes) as the primary analysis and reported ear-level results as a sensitivity analysis.

## Materials and Methods

### Study Design and Setting

This study is reported in accordance with the Strengthening the Reporting of Observational Studies in Epidemiology statement for observational studies [16]. A completed STROBE checklist and application memo are provided in online supplementary Table S1 (for all online suppl. material, see https://doi.org/10.1159/orl/aegag008). We conducted a single-center retrospective observational study at Yokohama City University (YCU) Medical Center, Japan.

### Participants

We identified consecutive patients diagnosed with EOM between April 2014 and April 2025 through a review of electronic medical records at the YCU Medical Center. All patients who met the diagnostic criteria for EOM during the study period were screened for eligibility. Patients were included if outcome assessments (severity score and audiometry) were available both before the index date and within a prespecified 12±2-month window after the index date (10–14 months). When multiple assessments were available, we selected the visit closest to the index date for the pre-index assessment and the visit closest to 12 months for the post-index assessment. EOM diagnosis followed the criteria proposed by Iino et al. [1]. Briefly, the major criterion was otitis media with effusion or chronic otitis media with eosinophil-dominant middle-ear effusion. A definitive diagnosis required fulfillment of the major criterion plus at least two of four minor criteria: highly viscous middle-ear effusion, resistance to conventional treatment for otitis media, comorbid bronchial asthma, and comorbid nasal polyposis. Exclusion criteria included missing pre- or post-index outcome data within the prespecified 12±2-month window and a diagnosis of eosinophilic granulomatosis with polyangiitis (formerly Churg-Strauss syndrome), as it has a distinct clinical course. In the present cohort, no patients were excluded because of missing outcome data within the prespecified window, but 1 patient was excluded because of eosinophilic granulomatosis with polyangiitis diagnosis. Based on the available medical records, no additional patients meeting the diagnostic criteria were excluded for reasons other than the prespecified exclusion criteria, including death, transfer of care, discontinuation of follow-up, or incomplete medical records. Comorbid ECRS was diagnosed and classified according to the Japanese Epidemiological Survey of Refractory Eosinophilic Chronic Rhinosinusitis criteria [17]. Rare disease designation refers to official registration of ECRS under Japan’s national program for designated intractable diseases. This program provides medical expense support for patients who meet predefined diagnostic and severity criteria. In the present study, rare disease designation was considered a marker of more severe or formally recognized ECRS rather than a diagnostic criterion for EOM.

### Index Date and Treatment Groups

Because this was a real-world cohort, the clinical context of treatment initiation differed by treatment strategy. Patients were assigned to the biologics group if they initiated dupilumab or mepolizumab for comorbid type 2 airway disease (asthma and/or ECRS) and had eligible pre- and post-index assessments anchored to that initiation. The index date for the biologics group was defined as the date of biologic therapy initiation. Patients were assigned to the non-biologics group if they were managed without biologics during the observation window and had eligible pre- and post-index assessments anchored to initiation of EOM-directed corticosteroid therapy. The index date for the non-biologics group was defined as the date EOM-directed corticosteroid therapy was initiated, operationalized as initiation of systemic corticosteroids and/or intratympanic steroid injections following diagnosis and the clinical decision to start steroid-based treatment. Biologic therapies were prescribed according to approved indications and physician judgment for comorbid airway disease; objective measures of asthma or ECRS severity were not uniformly available in this retrospective dataset.

The differential index date definitions were chosen to reflect the real-world clinical decision point for each treatment strategy. However, this design did not align patients at the same stage of the EOM disease trajectory. Patients in the biologics group may have had more established or refractory type 2 airway disease and prior EOM treatment exposure, whereas patients in the non-biologics group were indexed at the initiation of EOM-directed corticosteroid therapy. Accordingly, between-group comparisons were prespecified as exploratory and hypothesis-generating analyses rather than estimates of causal treatment effects.

### Treatments

Standard care included topical and/or intratympanic corticosteroids, with or without systemic corticosteroids, along with optimization of comorbid airway disease. Before biologic initiation, patients in the biologics group could receive conventional EOM treatments, including topical, intratympanic, or systemic corticosteroids, according to clinical need. In the biologics group, dupilumab or mepolizumab was prescribed for comorbid airway disease according to approved indications as adjunctive therapy during EOM management.

### Outcomes

Primary outcomes were (1) the EOM severity score and (2) air-conduction PTA. The EOM severity score was defined as a 0–10 composite score consisting of five items scored 0–2 points each: quantity of middle-ear effusion or otorrhea, condition of the middle-ear mucosa, frequency of intratympanic steroid instillation, frequency of systemic corticosteroid administration, and frequency of antibiotic use. This score was previously proposed to evaluate clinical severity and treatment response [18]. No established minimal clinically important difference has been defined for this score. Air-conduction PTA was calculated as the mean hearing threshold at 500, 1,000, 2,000, and 4,000 Hz. For the primary patient-level analysis, ear-specific values were averaged across ears when both ears were affected. Secondary outcomes included bone-conduction PTA (calculated using the same four frequencies). Pre-index measurements were compared with those obtained within the prespecified 12±2-month window after the index date (10–14 months). When a hearing threshold exceeded the audiometer limit, the measurable level plus 5 dB was recorded.

### Follow-Up Timing

The 12±2-month follow-up window was prespecified to evaluate medium-term clinical and audiometric outcomes while allowing practical variability in real-world outpatient follow-up schedules. This window was also selected to quantify systemic corticosteroid exposure and intratympanic steroid injection frequency over a clinically relevant 1-year period and to reduce heterogeneity in observation time. Post-index outcomes were assessed within this window (10–14 months after the index date). If multiple assessments were available within this window, the visit closest to 12 months was selected. In the analytic dataset, all selected post-index assessments occurred within 12–14 months (median: 12.5 months; IQR: 12.0–13.0), with no significant difference between groups (*p* = 0.945).

### Assessment of Corticosteroid Exposure

To quantify steroid-sparing effects, systemic corticosteroid exposure was calculated as the daily average dose (mg/day) of oral or intravenous corticosteroids (prednisolone equivalent). The cumulative systemic corticosteroid dose during each observation period was divided by the number of observed days (pre-index: up to 365 days before the index date; post-index: from the index date to the selected post-index assessment within 10–14 months). Local treatment burden was assessed as the mean frequency of intratympanic steroid injections (times per month) during the same observation periods. Because the index date for the non-biologics group was defined as initiation of EOM-directed corticosteroid therapy (systemic and/or intratympanic), pre-index EOM-directed systemic corticosteroid exposure and pre-index intratympanic injection frequency were not applicable by design and are reported accordingly.

### Statistical Analysis

Analyses were performed using R (R Foundation for Statistical Computing, Vienna, Austria). Given the small sample size and inability to reliably assess distributional assumptions, nonparametric methods were selected for the primary analyses. For the primary analysis, the patient was the unit of analysis, such that each patient contributed a single observation rather than treating bilateral ears from the same patient as statistically independent observations. For patients with bilateral EOM, ear-specific outcomes were averaged to generate patient-level outcomes; for unilateral disease, the affected ear was used. Within-group pre/post comparisons used the Wilcoxon signed rank test, and between-group comparisons used the Mann-Whitney U test for continuous variables. Fisher’s exact test was used for categorical variables because of small cell counts. To provide descriptive uncertainty estimates, 95% CIs for within-group change scores were estimated using nonparametric percentile bootstrapping with 5,000 patient-level resamples. Between-group differences in change scores were summarized using Hodges-Lehmann estimates with 95% CIs based on pairwise differences, corresponding to the Mann-Whitney U framework. Baseline characteristics were compared between groups and are presented in Table 1.

**Table 1.** Baseline characteristics

Characteristic	Total (*n* = 14 patients)	Biologics (*n* = 7)	Non-biologics (*n* = 7)	Between-group *p*
Age, years, mean±SD	57.29±9.25	55.86±10.32	58.71±8.62	0.798
Sex, *n* (%)	​	​	​	1.000
Male	3 (21.4)	2 (28.6)	1 (14.3)	​
Female	11 (78.6)	5 (71.4)	6 (85.7)	​
Comorbidities, *n* (%)	​	​	​	​
ECRS	14 (100.0)	7 (100.0)	7 (100.0)	1.000
Bronchial asthma	13 (92.9)	6 (85.7)	7 (100.0)	1.000
Rare disease designation, *n* (%)	7 (50.0)	7 (100.0)	0 (0.0)	<0.001
Affected ears, *n*	24	12	12	—
Laterality, *n* (%)	​	​	​	1.000
Bilateral	10 (71.4)	5 (71.4)	5 (71.4)	​
Unilateral	4 (28.6)	2 (28.6)	2 (28.6)	​
EOM phenotype (ears), *n* (%)	​	​	​	0.126
OME type	12 (50.0)	7 (58.3)	5 (41.7)	​
Perforated COM type	10 (41.7)	3 (25.0)	7 (58.3)	​
Granulation COM type	2 (8.3)	2 (16.7)	0 (0.0)	​

Values are presented as mean ± SD or *n* (%). Between-group comparisons were performed using the Mann-Whitney U test for continuous variables and Fisher’s exact test for categorical variables. Laterality is reported at the patient level, whereas EOM phenotype is reported at the ear level. Rare disease designation indicates official registration of ECRS under Japan’s government program for designated intractable diseases and was considered a marker of more severe or formally recognized ECRS, not a diagnostic criterion for EOM.

COM, chronic otitis media; ECRS, eosinophilic chronic rhinosinusitis; EOM, eosinophilic otitis media; OME, otitis media with effusion; SD, standard deviation.

To explore potential confounding, we additionally fitted patient-level linear regression models for change scores, adjusting for baseline outcome value and follow-up duration (months), with HC3 robust standard errors (online suppl. Table S2). A two-sided *p* < 0.05 was considered statistically significant. As a sensitivity analysis, ear-level outcomes were also analyzed and are reported in online supplementary Table S3.

Additional sensitivity analyses to address the differential index date definitions were considered. However, biologic initiation and the initiation of EOM-directed corticosteroid therapy represented inherently different clinical events, and no alternative index date could be applied uniformly across both groups without excluding substantial clinical information or introducing additional selection bias. Therefore, a sensitivity analysis based on alternative index dates was not performed. Instead, potential bias related to differential index date definitions was addressed by standardizing the post-index assessment window, prespecifying patient-level outcomes as the primary analysis, and performing exploratory regression adjustment for baseline outcome values and follow-up duration.

Propensity score-based methods, including matching, stratification, or inverse probability weighting, were considered to further address confounding by indication. However, these approaches were not performed because of the very small sample size and complete separation of rare disease designation by treatment group, which would render propensity score estimation statistically unstable and potentially misleading. Accordingly, adjusted analyses were limited to exploratory regression models adjusting for baseline outcome values and follow-up duration.

Because this was a pilot retrospective cohort study of a rare disease, no formal a priori sample size calculation was performed. To inform the design of future studies, we performed exploratory sample size estimation based on the observed between-group differences in patient-level change scores for EOM severity score and air-conduction PTA. Sample size estimates were calculated for two-group comparisons with equal group sizes, assuming a two-sided α of 0.05 and 80% power. These estimates were intended only for future study planning and were not used to support confirmatory inference in the present study. To explore heterogeneity within the biologics group, anonymized individual patient-level outcomes according to biologic agent were summarized descriptively.

## Results

### Participant Characteristics

A total of 14 patients with EOM were included (three men and 11 women). Seven patients received biologic therapy (dupilumab: *n* = 6; mepolizumab: *n* = 1) and seven received standard care without biologics. Individual patient-level outcomes in the biologics group according to biologic agent are summarized in online supplementary Table S4. Post-index outcomes were assessed within a prespecified 12±2-month window after the index date (10–14 months), selecting the visit closest to 12 months when multiple assessments were available. Baseline characteristics are summarized in Table 1. Rare disease designation differed significantly between groups (biologics 7/7 [100.0%] vs. non-biologics 0/7 [0.0%]; *p* < 0.001). Age and sex distributions were similar between groups (age: 55.86 ± 10.32 vs. 58.71 ± 8.62 years; *p* = 0.798; male: 2/7 [28.6%] vs. 1/7 [14.3%]; *p* = 1.000), and EOM phenotype distribution at the ear level did not differ significantly (*p* = 0.126).

### Changes in Severity Score and Hearing (Primary Patient-Level Analysis)

Patient-level pre-post changes in EOM severity score and hearing outcomes are summarized in Table 2. EOM severity scores improved in both groups (Fig. 1a). At baseline, mean severity scores were 4.9 ± 1.7 in the biologics group and 5.7 ± 1.1 in the non-biologics group (between-group *p* = 0.361). Post-index severity scores were lower in the biologics group (0.3 ± 0.5) than in the non-biologics group (3.5 ± 1.6; between-group *p* = 0.003). However, the between-group difference in change score (Δ) did not reach statistical significance (*p* = 0.077).

**Table 2.** Patient-level pre-post changes in EOM severity score and hearing outcomes (primary analysis)

Outcome	Group	Pre-index	Post-index	Δ (post-pre)	Within-group *p*	Between-group *p* for Δ	Between-group difference in Δ, Hodges-Lehmann estimate (95% CI)
EOM severity score (0–10)	Biologics	4.93±1.69	0.29±0.49	−4.64 (−5.79 to −3.50)	0.016	0.077	−2.0 (−5.0 to 0.0)
	Non-biologics	5.71±1.11	3.50±1.61	−2.21 (−3.57 to −0.64)	0.044	​	​
Air-conduction PTA, dB HL	Biologics	39.55±19.05	28.04±16.85	−11.52 (−18.84 to −4.29)	0.031	0.249	−5.6 (−21.9 to 12.5)
	Non-biologics	55.00±19.03	50.54±23.35	−4.46 (−15.18 to 5.54)	0.812	​	​
Bone-conduction PTA, dB HL	Biologics	21.16±11.48	20.89±13.48	−0.27 (−2.68 to 1.96)	0.916	0.847	−0.6 (−10.0 to 6.9)
	Non-biologics	32.86±16.07	33.30±18.63	0.45 (−5.80 to 6.88)	1.000	​	​

Values are patient-level outcomes. Pre- and post-index values are presented as mean ± SD. Change scores (Δ) were calculated as post-index minus pre-index and are presented as mean (95% CI). For patients with bilateral EOM, ear-specific values were averaged to generate patient-level values. Negative Δ values indicate improvement for EOM severity score and PTA. PTA was calculated as the mean hearing threshold at 500, 1,000, 2,000, and 4,000 Hz. Within-group comparisons used the Wilcoxon signed-rank test; between-group comparisons of Δ used the Mann-Whitney U test. The 95% CIs for within-group change scores were estimated using nonparametric percentile bootstrapping with 5,000 patient-level resamples. Between-group differences in Δ are presented as Hodges-Lehmann estimates with 95% CIs, calculated as the median of all pairwise between-group differences in Δ and corresponding to the Mann-Whitney U framework. All analyses are exploratory because of the small sample size.

CI, confidence interval; EOM, eosinophilic otitis media; PTA, pure-tone average; SD, standard deviation.

**Fig. 1.**
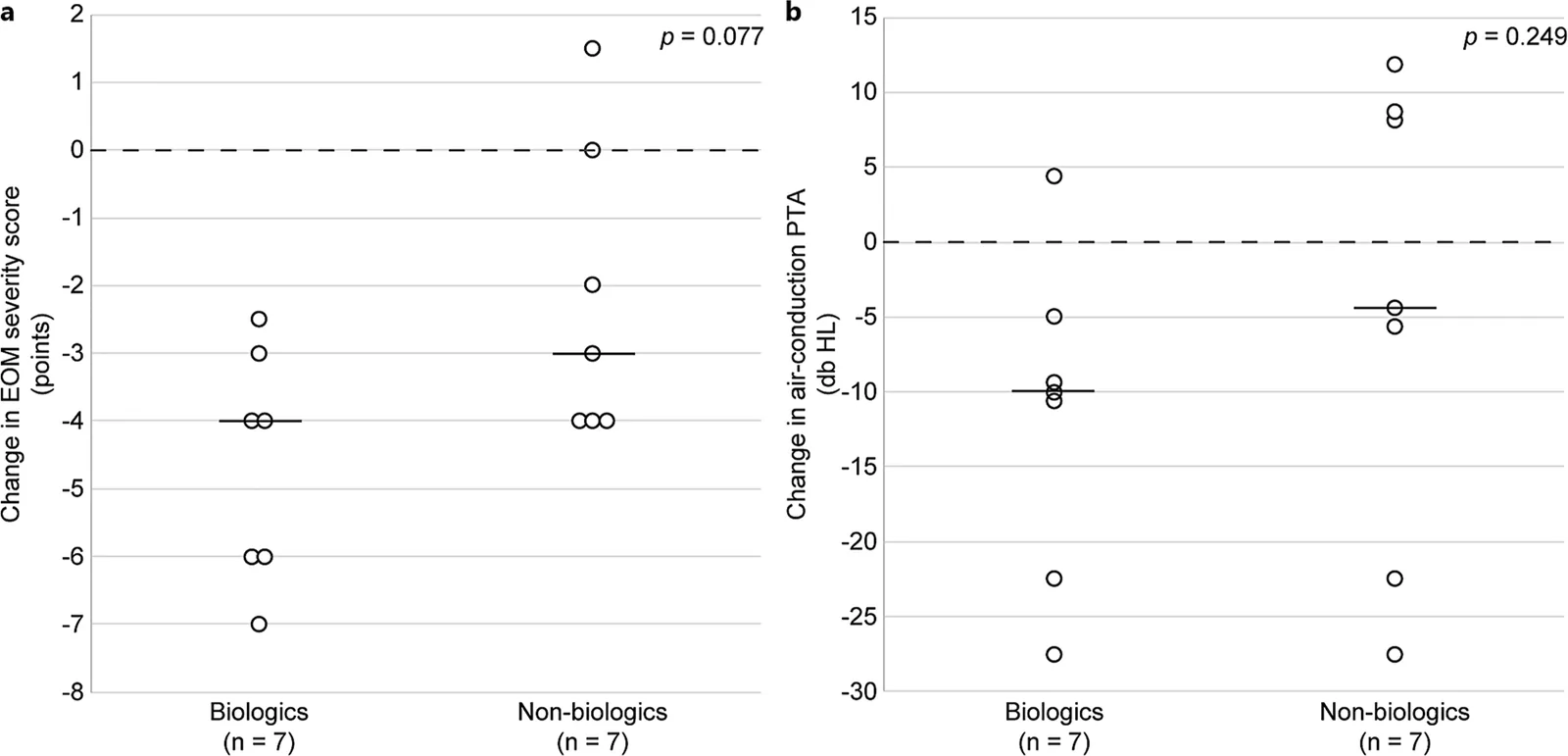
Patient-level changes in EOM severity score and air-conduction pure-tone average (PTA). Change in EOM severity score (**a**) and change in air-conduction PTA (500/1,000/2,000/4,000 Hz) (**b**) from pre-index to post-index assessment. Each dot represents 1 patient. For patients with bilateral EOM, ear-specific values were averaged to derive patient-level outcomes; for unilateral disease, the affected ear was used. Change was calculated as the post-index value minus the pre-index value. Horizontal bars indicate medians. The dashed horizontal line indicates no change. Values below zero indicate improvement for both outcomes. The post-index assessment was selected as the visit closest to 12 months within the prespecified 10- to 14-month window. *p* values indicate between-group comparisons of change scores.

For hearing outcomes, baseline air-conduction PTA was 39.6 ± 19.0 dB in the biologics group and 55.0 ± 19.0 dB in the non-biologics group (between-group *p* = 0.159). Air-conduction PTA improved within the biologics group (post-index 28.0 ± 16.8 dB; within-group *p* = 0.031), whereas improvement within the non-biologics group was not statistically significant (post-index 50.5 ± 23.4 dB; within-group *p* = 0.812). Between-group differences in change in air-conduction PTA were not statistically significant (Fig. 1b; Table 2) (*p* = 0.249). Frequency-specific air-conduction thresholds at 125–8,000 Hz before and after the index date are shown descriptively in online supplementary Figure S1. Changes in bone-conduction PTA were not statistically significant within either group (Table 2).

### Corticosteroid Exposure and Local Treatment Burden

Systemic corticosteroid exposure decreased in the biologics group from 1.79 ± 1.49 mg/day pre-index to 0.00 ± 0.00 mg/day post-index (within-group *p* = 0.016), with complete discontinuation in all biologics-treated patients. Because the index date for the non-biologics group was defined as initiation of EOM-directed corticosteroid therapy, pre-index EOM-directed systemic steroid exposure was not applicable by design; therefore, within-group pre-post comparisons for systemic steroid exposure were not performed in that group. Post-index systemic corticosteroid exposure remained 1.35 ± 1.55 mg/day, which was higher than that in the biologics group (between-group *p* = 0.011) (Fig. 2a; Table 3).

**Fig. 2.**
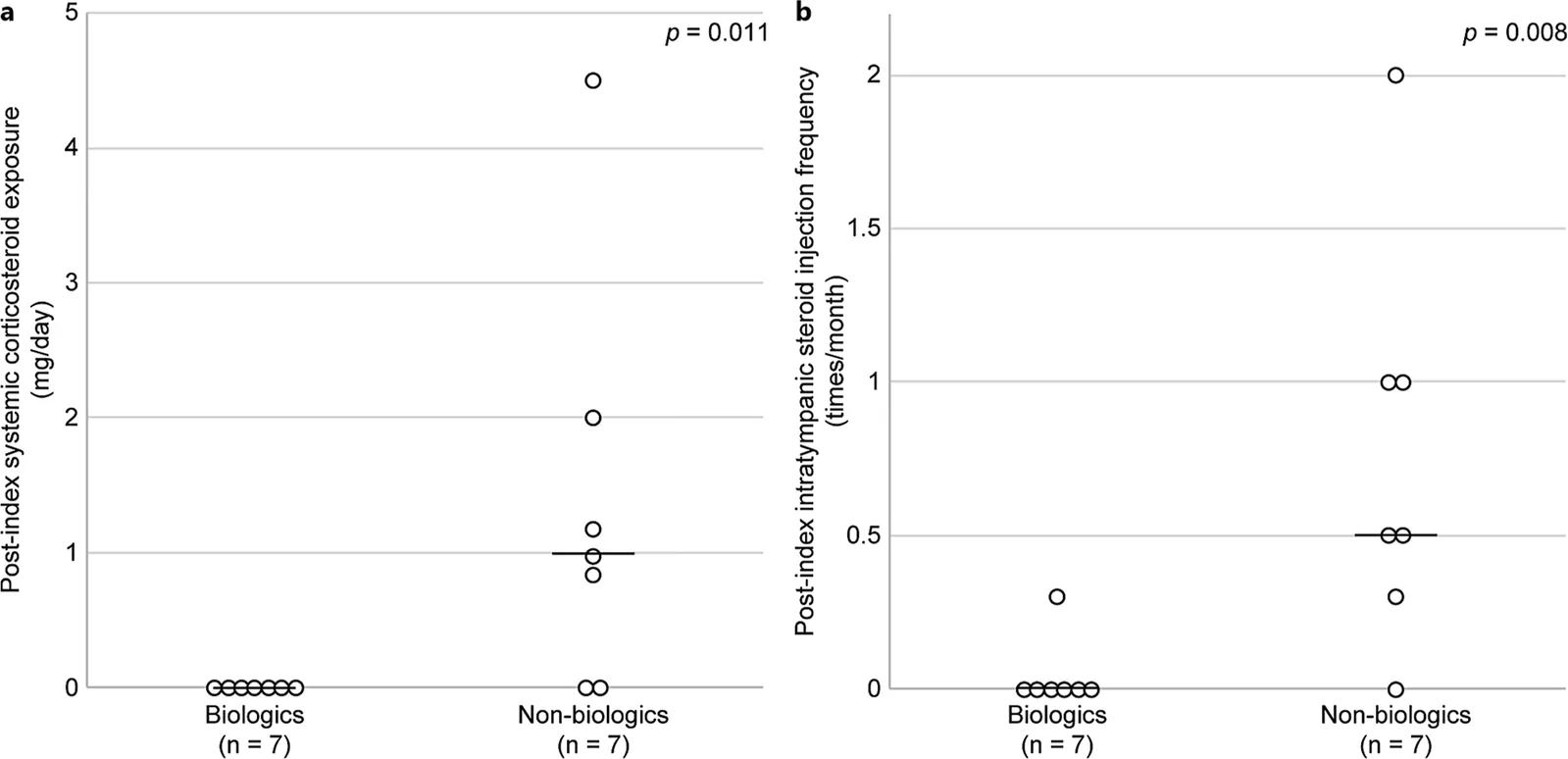
Post-index corticosteroid exposure and local treatment burden. Post-index systemic corticosteroid exposure, expressed as the average daily prednisolone-equivalent dose (**a**), and post-index intratympanic steroid injection frequency (**b**). Each dot represents 1 patient, and horizontal bars indicate medians. Values were calculated from the index date to the selected post-index assessment within the prespecified 10- to 14-month window. Pre-index values in the non-biologics group are not shown because, by design, the index date in this group was defined as initiation of EOM-directed corticosteroid therapy. Lower values indicate lower systemic corticosteroid exposure and lower local treatment burden. *p* values indicate between-group comparisons of post-index values.

**Table 3.** Patient-level systemic corticosteroid exposure and intratympanic steroid injection frequency

Outcome	Biologics (*n* = 7 patients)	Non-biologics (*n* = 7 patients)	Between-group *p*
Systemic corticosteroids, mg/day			
Pre-index, mean±SD	1.79±1.49	N/A (by design)	–
Post-index, mean±SD	0.00±0.00	1.35±1.55	0.011
Within-group *p* (Wilcoxon)	0.016	N/A	–
Intratympanic steroid injections (times/month)			
Pre-index, mean±SD	0.82±0.87	N/A (by design)	–
Post-index, mean±SD	0.04±0.11	0.76±0.66	0.008
Within-group *p* (Wilcoxon)	0.043	N/A	–

Values are presented as mean ± SD unless otherwise indicated. Systemic corticosteroid exposure was calculated as the average daily oral or intravenous corticosteroid dose and is expressed as prednisolone-equivalent milligrams per day. Intratympanic steroid injection frequency was calculated as the average number of injections per month. In the non-biologics group, pre-index EOM-directed systemic corticosteroid exposure and intratympanic steroid injection frequency were not applicable by design because the index date was defined as initiation of EOM-directed corticosteroid therapy. Between-group comparisons were performed for post-index values using the Mann-Whitney U test. Within-group pre-post comparisons were performed using the Wilcoxon signed-rank test in the biologics group only. All tests were two-sided.

EOM, eosinophilic otitis media; N/A, not applicable; SD, standard deviation.

The frequency of intratympanic steroid injections decreased in the biologics group from 0.82 ± 0.87 to 0.04 ± 0.11 times/month (within-group *p* = 0.043). Pre-index intratympanic injection frequency in the non-biologics group was also not applicable by design for the same reason. Post-index injection frequency was 0.76 ± 0.66 times/month, which was higher than that in the biologics group (between-group *p* = 0.008) (Fig. 2b; Table 3).

### Sensitivity Analysis (Ear-Level)

Ear-level analyses showed patterns generally consistent with the patient-level results and are summarized in online supplementary Table S3. Because ear-level analyses treat ears as independent observations and do not account for within-patient correlation, the corresponding *p* values should be regarded as exploratory.

### Exploratory Regression Adjusting for Follow-Up Duration

In exploratory patient-level linear regression models adjusting for the baseline outcome value and follow-up duration (months) and using HC3 robust standard errors, biologic exposure was associated with a greater reduction in severity score (β = −3.27 points; 95% confidence interval [CI]: −4.73 to −1.81; *p* < 0.001) and greater improvement in air-conduction PTA (β = −10.35 dB; 95% CI: −16.21 to −4.49; *p* = 0.001) (online suppl. Table S2). Follow-up duration was included only to account for minor variability within the prespecified assessment window. Because of the narrow follow-up range and small sample size, these regression results should be considered exploratory.

### Exploratory Sample Size Estimation for Future Studies

Exploratory sample size estimation based on the observed patient-level change scores suggested that approximately 12 patients per group would be required to detect the observed between-group difference in EOM severity score change with 80% power at a two-sided α of 0.05. In contrast, approximately 58 patients per group would be required to detect the observed between-group difference in air-conduction PTA change under the same assumptions. These estimates, summarized in online supplementary Table S5, are provided only for future study planning because they were derived from a small pilot cohort.

## Discussion

In this pilot single-center retrospective cohort of patients with EOM, biologic therapy with dupilumab or mepolizumab for comorbid type 2 airway disease was associated with a reduced corticosteroid burden and lower post-index EOM severity. We conducted this study to address a clinically relevant gap: whether biologic therapy prescribed for comorbid type 2 airway disease is associated with changes in EOM severity, hearing outcomes, and quantifiable corticosteroid burden in real-world practice. Although air-conduction PTA improved within the biologics group, the between-group difference in PTA change was not statistically significant. Therefore, the hearing findings should be considered preliminary and hypothesis-generating rather than evidence of a definitive hearing benefit attributable to biologic therapy. The observed change in EOM severity score may nevertheless be clinically meaningful. No established minimal clinically important difference has been defined for this score; however, the reduction observed in the biologics group reflected improvements across multiple clinically relevant domains, including middle-ear effusion or otorrhea, middle-ear mucosal condition, intratympanic steroid use, and systemic corticosteroid use. In particular, complete discontinuation of systemic corticosteroids and the near-elimination of intratympanic steroid injections represent clinically relevant reductions in treatment burden. Accordingly, the changes in the severity score should be interpreted not merely as a numerical change but as a composite improvement in disease activity and treatment intensity. However, because the severity score includes treatment-intensity components, part of the observed improvement may reflect a reduction in treatment burden rather than inflammatory disease activity alone. These findings add to the emerging real-world evidence suggesting potential benefits of type 2-targeted biologic therapies for refractory EOM [3, 6–9, 13–15].

These findings align with the mechanistic rationale that type 2-targeted biologics may attenuate eosinophilic inflammation across the unified airway and middle ear. Dupilumab inhibits IL-4/IL-13 signaling via IL-4Rα, whereas mepolizumab targets IL-5; both mechanisms reduce eosinophilic inflammation and mucus production. IL-13 has also been implicated in eosinophilic airway-middle ear disease in patients with severe asthma [19]. However, because biologic selection and initiation were not randomized and were driven by management of comorbid airway disease, our results should be interpreted as associations rather than evidence of comparative efficacy between strategies.

Another limitation is the heterogeneity of biologic therapy. The biologics group included both dupilumab and mepolizumab, which target different components of type 2 inflammation. Although both agents may plausibly improve eosinophilic inflammation, their effects on EOM severity, middle-ear findings, corticosteroid requirements, and hearing outcomes may not be equivalent. To clarify the direction and variability of responses, we added anonymized individual patient-level outcomes by biologic agent in online supplementary Table S4. However, because only 1 patient received mepolizumab, no formal agent-specific comparison was possible, and the biologics group should be considered as a heterogeneous exposure category rather than as evidence for a uniform class effect.

Baseline imbalance and confounding by indication are major limitations of this study. Baseline EOM severity score and air-conduction PTA differed between groups, and rare disease designation for ECRS was present in all biologics-treated patients but in none of the non-biologics patients. This pattern indicates substantial differences in disease severity, healthcare pathways, eligibility for advanced type 2-targeted therapy, and access to biologic treatment. Therefore, biologic exposure was not simply a treatment variable but also a marker of a distinct clinical background. These differences may have influenced both the likelihood of receiving biologic therapy and the subsequent clinical course, resulting in substantial confounding by indication.

Although we performed exploratory regression adjustment for baseline outcome values and follow-up duration, this approach cannot fully address residual or unmeasured confounding in such a small cohort. Important potential confounders, including objective measures of asthma or ECRS severity, duration of EOM, prior cumulative corticosteroid exposure, and previous local treatments, were not uniformly available in the retrospective dataset. We considered propensity score-based approaches; however, the very small sample size and complete separation of rare disease designation by treatment group made propensity score estimation statistically unstable and potentially misleading. Accordingly, we did not perform propensity score matching, stratification, or inverse probability weighting. These limitations further support interpreting the observed associations, including between-group comparisons, as descriptive and hypothesis-generating rather than evidence of comparative efficacy.

A key methodological limitation is that the index date reflected different clinical contexts across groups. In the biologics group, the index date was defined as initiation of biologic therapy for comorbid type 2 airway disease, whereas in the non-biologics group it was defined as initiation of EOM-directed corticosteroid therapy. Therefore, the two groups may not have been aligned at the same stage of EOM disease trajectory. Patients receiving biologics may have had more established, refractory, or previously treated type 2 airway disease, whereas patients in the non-biologics group may have been captured closer to the initial decision to initiate EOM-directed steroid treatment. This structural asymmetry may introduce bias through differences in disease chronicity, prior treatment exposure, background airway disease severity, and healthcare pathways. It may also contribute to regression-to-the-mean effects; patients may have been more likely to initiate or escalate treatment when symptoms, otorrhea, middle-ear inflammation, or hearing thresholds were relatively severe, and subsequent improvement may partly reflect natural fluctuation toward the patient’s usual disease state rather than the treatment itself.

Because no alternative index date could be uniformly applied across both groups without introducing additional selection bias, we did not perform a separate index-date sensitivity analysis. Instead, we standardized the post-index assessment window, used patient-level outcomes as the primary analysis, and performed exploratory regression adjustment for baseline outcome values and follow-up duration. Because follow-up duration varied only within a narrow range, its regression coefficient should be regarded as an unstable adjustment term rather than as a clinically meaningful time effect. Nevertheless, these approaches cannot fully eliminate bias arising from differential disease trajectory, and the findings should therefore be considered as preliminary associations rather than causal estimates of biologic efficacy. We sought to mitigate clustering-related concerns by prespecifying patient-level outcomes (ear-averaged) as the primary analysis and reporting ear-level analyses only as sensitivity analyses. Because the ear-level analyses treated ears as independent observations and did not account for within-patient correlation, these findings should be regarded as exploratory supportive analyses rather than independent confirmatory evidence.

The hearing outcomes require particular caution. Although air-conduction PTA improved significantly within the biologics group, the between-group difference in PTA change was not statistically significant. Therefore, the present data do not establish that biologic therapy provides superior hearing improvement compared with non-biologic management. The within-group improvement may reflect treatment effects, natural disease fluctuation, regression-to-the-mean, differences in baseline hearing status, or other unmeasured clinical factors. Accordingly, the hearing findings should be regarded as hypothesis-generating and should be confirmed in larger prospective studies with standardized audiometric follow-up.

The exploratory sample size estimation further illustrates the implications of these findings for future research. Based on the observed patient-level change scores, a future study would require approximately 12 patients per group to detect the observed between-group difference in EOM severity score change with 80% power at a two-sided α of 0.05. In contrast, approximately 58 patients per group would be required to detect the observed between-group difference in air-conduction PTA change. These estimates suggest that EOM severity score and corticosteroid burden may be more feasible primary endpoints for future multicenter studies, whereas audiometric outcomes may require substantially larger cohorts or may be more appropriately considered key secondary outcomes.

Our findings regarding systemic steroid discontinuation are clinically relevant given the well-described risks associated with systemic corticosteroid exposure, even with relatively short courses, and the potential for adrenal insufficiency with repeated or prolonged use [5, 20]. Nevertheless, larger prospective studies with standardized assessment time points and predefined steroid exposure windows are needed to establish comparative effectiveness and identify which patients are most likely to benefit from biologic therapy [3, 15].

## Conclusions

In this pilot retrospective cohort, biologic therapy with dupilumab or mepolizumab for comorbid type 2 airway disease was associated with a reduced corticosteroid burden and lower post-index EOM severity in patients with EOM. Although air-conduction PTA improved within the biologics group, the between-group difference in PTA change was not statistically significant. These findings should be interpreted as preliminary associations in the context of the small sample size, baseline imbalance, potential confounding by indication, nonrandomized treatment allocation, and differential index date definitions that may have captured patients at different stages of EOM disease trajectory.

## Acknowledgments

We would like to thank Editage (www.editage.jp) for English language editing.

## Statement of Ethics

This study was conducted at Yokohama City University Medical Center and approved by the Yokohama City University Certified Institutional Review Board (approval no: F260200013). All procedures complied with the principles of the Declaration of Helsinki. The requirement for informed consent was waived by the Institutional Review Board of Yokohama City University Medical Center due to the retrospective nature of the study. An opt-out method was used to allow patients to decline participation.

## Conflict of Interest Statement

The authors have no conflicts of interest to declare.

## Funding Sources

This study was not supported by any sponsor or funder.

## Author Contributions

T.W. contributed to conceptualization, study design, investigation, data curation, formal analysis, and writing – original draft. Y. Arai contributed to conceptualization. Y. Aizawa and K.S. contributed to data curation. H.H. contributed to formal analysis. N.O. contributed to methodology, formal analysis, and supervision. All authors reviewed and approved the final manuscript.

## Data Availability

All data generated or analyzed during this study are included in this published article and its online supplementary information files. Further inquiries can be directed to the corresponding author.
